# Cholesteryl ester transfer protein inhibition with obicetrapib is associated with attenuated decline in kidney function in patients at high cardiovascular risk: Post hoc pooled results from the BROADWAY and BROOKLYN trials

**DOI:** 10.1016/j.ajpc.2026.101532

**Published:** 2026-03-24

**Authors:** Liffert Vogt, Michael H. Davidson, Michael Szarek, Douglas Kling, Mary R. Dicklin, Danielle Curcio, Marc Ditmarsch, Andrew Hsieh, Douglas L. Wicks, Nancy Ortiz, John J.P. Kastelein

**Affiliations:** a Section of Nephrology, Department of Internal Medicine, Academic Medical Center, University of Amsterdam, the Netherlands; b NewAmsterdam Pharma, Naarden, the Netherlands; c University of Colorado Anschutz Medical Campus and CPC Clinical Research, Aurora, CO, USA; d Mount Sinai Fuster Heart Hospital, Icahn School of Medicine at Mount Sinai, NY, NY, USA; e State University of New York Downstate School of Public Health, Brooklyn, NY, USA; f Midwest Biomedical Research, Addison, IL, USA

**Keywords:** Cholesteryl ester transfer protein, Obicetrapib, High-density lipoprotein function, Highdensity, Lipoprotein cholesterol, Chronic kidney disease, Atherosclerotic, Cardiovascular disease

## Abstract

**Background:**

Patients with cardiovascular disease have increased risk of chronic kidney disease progression. Low levels of high-density lipoprotein (HDL) or HDL dysfunction have been implicated in progressive deterioration of renal function. We investigated the impact on renal function of obicetrapib, a cholesteryl ester transfer protein (CETP) inhibitor that raises apolipoprotein A1 and HDL cholesterol (HDL-C) and improves HDL function.

**Methods:**

BROADWAY (*N* = 2530) and BROOKLYN (*N* = 354) were double-blind, placebo-controlled trials of patients with heterozygous familial hypercholesterolemia and/or established atherosclerotic cardiovascular disease taking maximally tolerated lipid-lowering therapy assigned to 365-day treatment with the novel CETP inhibitor, obicetrapib 10 mg/d, or placebo. In a post hoc pooled trial analysis of randomized participants with at least 1 post-baseline renal assessment (estimated glomerular filtration rate [eGFR] *n* = 2832; urine albumin-to-creatinine ratio [UACR] *n* = 1897), renal function and its association with HDL-C were evaluated.

**Results:**

Participants in BROADWAY and BROOKLYN had mean (±SD) baseline eGFR 84.4 ± 17.9 and 91.8 ± 17.8 mL/min/1.73 m² and median albumin-to-creatinine ratio 11.0 and 8.0 mg/g, respectively. Obicetrapib attenuated kidney function decline vs placebo (mean difference = 0.67 mL/min/1.73 m², nominal *P* = 0.02); on-treatment analysis strengthened this signal (0.82; nominal *P* = 0.0139). Obicetrapib-treated patients had nominally fewer cases of eGFR <15 mL/min/1.73 m^2^ (0 vs 0.2 %),≥40 % eGFR declines (1.3 vs 1.9 %), and a renal events composite (1.9 vs 3.0 %; hazard ratio 0.64, nominal *P* = 0.08). Higher achieved HDL-C was associated with lower renal composite event risk (spline nominal *P* < 0.0001), independent of baseline HDL-C and eGFR. Annualized percent change in UACR was not significantly different between groups.

**Conclusion:**

In patients at high cardiovascular risk, CETP inhibition with obicetrapib may attenuate kidney function decline, potentially through its HDL-raising effects.

## 1 Introduction

Chronic kidney disease (CKD) is associated with increased risk of cardiovascular disease (CVD); in fact, CVD is a leading cause of mortality in persons with CKD, particularly those with severe renal dysfunction [1,2]. CKD has kidney-specific risk factors as well as risk factors that overlap with those of CVD, e.g., hypertension, diabetes, and dyslipidemia [1]. An examination of National Health and Nutrition Examination Survey data indicated 45.5 % of individuals with CKD stage 1 and up to 67.8 % with CKD stage 4 have dyslipidemia [3]. Dyslipidemia in CKD involves higher levels of triglycerides and triglyceride-rich lipoproteins and small, dense low-density lipoprotein (LDL), as well as a lower level of high-density lipoprotein cholesterol (HDL-C) [4]. Low HDL-C in patients with CKD is associated with adverse cardiovascular and renal outcomes [5]. Not only is the concentration of HDL-C reduced with CKD, but the composition of HDL is also modified, namely apolipoprotein (apo) A1 and apoA2 are diminished, and the cardiovascular-renal protective functions of HDL, including its cholesterol efflux capacity, anti-inflammatory, antioxidant, and vasodilatory properties may be disordered [5,6].

Cholesteryl ester transfer protein (CETP) inhibitors dramatically increase HDL-C by impairing the transfer of cholesteryl esters from HDL to apoB-containing lipoprotein particles. In a drug target Mendelian randomization analysis, lower CETP concentration was associated with lower risk of CKD (odds ratio 0.94; 95 % CI 0.91, 0.97) [7]. Obicetrapib is a novel CETP inhibitor that was shown in a meta-analysis of 7 trials to substantially reduce LDL-C (−37.2 %), apoB (−24.7 %), lipoprotein(a) (−37.2 %), non-HDL-C (−31.9 %), and risk of new-onset diabetes (-12 %), and increase HDL-C (+142 %) and apoA1 (+52.8 %), compared with placebo [8]. Obicetrapib has also been suggested to restore the functionality of HDL which may improve kidney health, although further research is needed [4,9]. The impact on kidney function of available lipid-lowering therapies is not fully understood [10]. The effect of obicetrapib on atherosclerotic cardiovascular events (ASCVD) events is under investigation in the Cardiovascular Outcome Study to Evaluate the Effect of Obicetrapib in Patients with Cardiovascular Disease (PREVAIL; NCT05202509). In this article we describe an exploratory, post hoc pooled analysis of renal function and its relation to HDL-C levels among participants in the Randomized Study to Evaluate the Effect of Obicetrapib on Top of Maximum Tolerated Lipid-Modifying Therapies (BROADWAY) and the Evaluate the Effect of Obicetrapib in Patients with Heterozygous Familial Hypercholesterolemia (HeFH) on Top of Maximum Tolerated Lipid-Modifying Therapies (BROOKLYN) to inform the clinical potential of the use of obicetrapib in patients with diminished renal function.

## 2 Methods

### 2.1 Study design

BROADWAY (NCT05142722) and BROOKLYN (NCT05425745) were multinational, placebo-controlled, double-blind, randomized Phase III trials examining the efficacy, safety, and tolerability of 10 mg daily obicetrapib, compared to placebo, taken for 365 days as an adjunct to dietary intervention and maximally tolerated lipid-modifying therapies by participants with a history of ASCVD and/or HeFH whose LDL-C was not adequately controlled. Details of BROADWAY and BROOKLYN have been published previously [[11], [12], [13]]. Ethical approval for BROADWAY and BROOKLYN were obtained from the relevant ethical review boards for each clinical research site where these trials were conducted [11,12].

### 2.2 Participants

Participants randomized in both trials were ≥18 years of age, on a maximally tolerated lipid-modifying therapy as an adjunct to a lipid-lowering dietary pattern and other lifestyle modifications, and had estimated glomerular filtration rate (eGFR) ≥30 mL/min/1.73 m^2^. Maximally tolerated lipid-modifying therapy included statin at a stable dose, ezetimibe (with or without maximally tolerated statin), and/or bempedoic acid with maximally tolerated statin for at least 8 weeks prior to screening, or proprotein convertase subtilisin/kexin type 9 inhibitor therapy (with or without other lipid-modifying therapy) for at least 4 doses prior to screening.

Participants in BROADWAY had a history of ASCVD and/or underlying HeFH (determined by genotyping or according to Simon Broome or World Health Organization-Dutch Lipid Clinic Network criteria), and fasting serum LDL-*C* ≥ 55 mg/dL to <100 mg/dL OR non-HDL-*C* ≥ 85 mg/dL to <130 mg/dL (for patients with at least 1 ASCVD risk enhancer) OR had fasting serum LDL-*C* ≥ 100 mg/dL OR non-HDL-*C* ≥ 130 mg/dL. BROADWAY participants were also required to have fasting serum triglycerides <500 mg/dL. Participants in BROOKLYN had a history of HeFH, fasting serum LDL-*C* ≥ 70 mg/dL, and fasting serum triglycerides <400 mg/dL.

### 2.3 Trial procedures

Participants in BROADWAY (*N* = 2532) and BROOKLYN (*N* = 354) were randomized 2:1 to receive obicetrapib 10 mg or placebo daily for 365 days and completed clinic visits at randomization, approximately 1, 3, 6, 9,and 12 months after randomization, and approximately 1 month after last dose, when fasting lipoprotein lipids and laboratory investigations – including those for estimated glomerular filtration rate (eGFR), calculated with the 2021 CKD EPI formula – were measured and adverse events (AEs), events of special interest (including renal abnormalities), and concomitant medication use were assessed. AEs were categorized by primary system organ class and preferred term as coded using the Medical Dictionary for Regulatory Activities category designations. Urine albumin-to-creatinine ratio (UACR) was assessed on the above schedule in BROOKLYN and at 12 months in BROADWAY. Apart from urine dipstick, which was performed on-site, laboratory analyses were performed by Medpace Research Laboratories. Further details and baseline characteristic parameters of this specific post hoc pooled BROOKLYN/BROADWAY analysis are found within Supplemental Table 1 of this report.

### 2.4 Statistical analyses

This post hoc pooled analysis of the BROADWAY and BROOKLYN trials included the intent-to-treat populations (all randomized participants) with at least 1 post-baseline eGFR assessment (*n* = 2832) (or at least 1 post-baseline urine albumin-to-creatinine ratio [UACR] measurement for UACR assessments; *n* = 1897). Between treatment group comparisons of the annualized change in eGFR and the annualized percent change in UACR were performed using repeated measures mixed effects models with random intercept and unstructured covariance matrix and terms for baseline eGFR (for the analysis of eGFR change) or UACR (for the analysis of UACR change), treatment assignment, study, time, and the interaction between treatment assignment and time. Missing values were not imputed. An on-treatment assessment of eGFR was similarly conducted that excluded eGFR assessments after the last dose of study drug. Subgroups defined by baseline eGFR <60 vs. ≥60 mL/min/1.73 m^2^ were assessed by adding an interaction term to the eGFR change model. A treatment comparison of time from randomization to a renal event composite – consisting of death due to cardiovascular or renal causes, eGFR <15 mL/min/1.73 m^2^ post-baseline, ≥40 % decline in eGFR from baseline, kidney transplant, or dialysis initiation – through end of follow-up (median (Q1, Q3) = 365 (362, 370), maximum 528 days) was assessed using a proportional hazards model with terms for baseline eGFR, treatment assignment, and study. The laboratory-based eGFR components of the composite did not require >1 measurement meeting the event criteria, and none of the components were adjudicated. Participants without an event were censored at the last eGFR assessment. The proportional hazards assumption was evaluated by examining the Schoenfeld residual plot and log–log plot; there was no evidence of deviation from the assumption.

Time-weighted achieved HDL-C concentration was related to the absolute risk of the renal composite through 1 year with a restricted cubic spline from a proportional hazard model with knots at the 33^rd^ and 67^th^ percentiles and plotted from the approximate 1^st^ to 99^th^ percentiles. In addition to time-weighted achieved HDL-C, the model included terms for baseline HDL-C, baseline eGFR, baseline LDL-C, time-weighted achieved LDL-C, study, age, sex, history of diabetes, history of hypertension, baseline statin intensity, and randomized treatment assignment. The time-weighted concentration for each participant was a single value calculated as the area under the curve using all available values before the participant’s event date or date of right censoring for the composite event divided by the number of days from baseline to the last value. The area under the curve was calculated using the linear trapezoidal rule accounting for the number of days from baseline for each assessment. Missing values were not imputed. All *P* values reported are considered nominal and no adjustment for multiple testing was performed.

## 3 Results

**Fig. 1:**
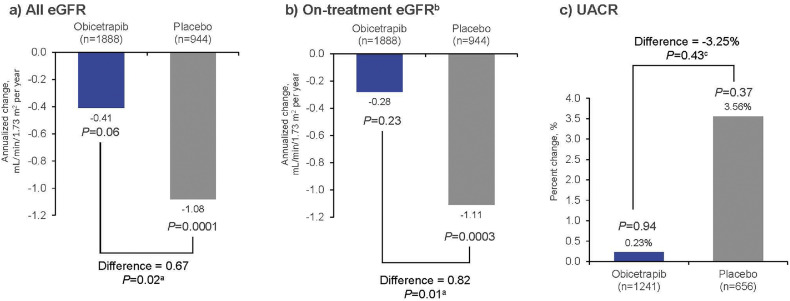
(Replaced Original Data Depiction as former Table). Abbreviations: eGFR, estimated glomerular filtration rate; UACR, urine albumin-to-creatinine ratio. Note: UACR percent changes based on log-transformed values. ^a^Nominal *P* values. ^b^Excluded eGFR assessments after last dose of study drug. ^c^Obicetrapib (*n* = 1241), placebo (*n* = 656), and difference (*n* = 1897).

Baseline demographics, blood pressure, and concomitant reno-protective medications (angiotensin-converting enzyme inhibitors, angiotensin receptor blockers, sodium-glucose cotransporter 2 inhibitors) were well-balanced in BROADWAY and BROOKLYN and remained stable throughout the trials [[11], [12], [13]]. The treatment comparisons of the annualized change in eGFR and annualized percent change in UACR in the pooled BROADWAY and BROOKLYN trials are shown in Table Fig. 1.

**Fig. 2:**
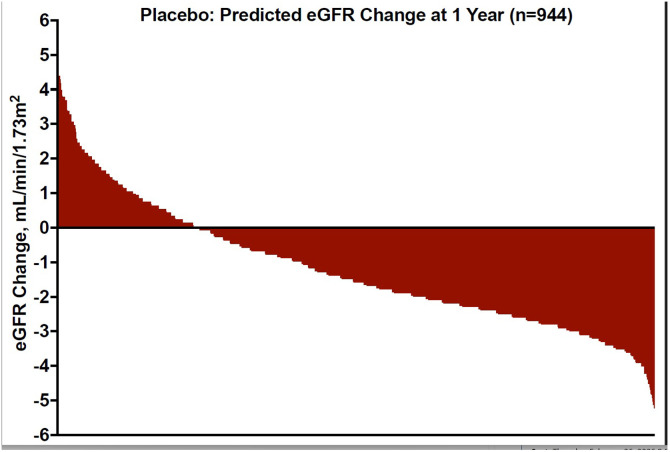
Placebo: Predicted eGFR Change a 1 year (*n* = 944).

**Fig. 3:**
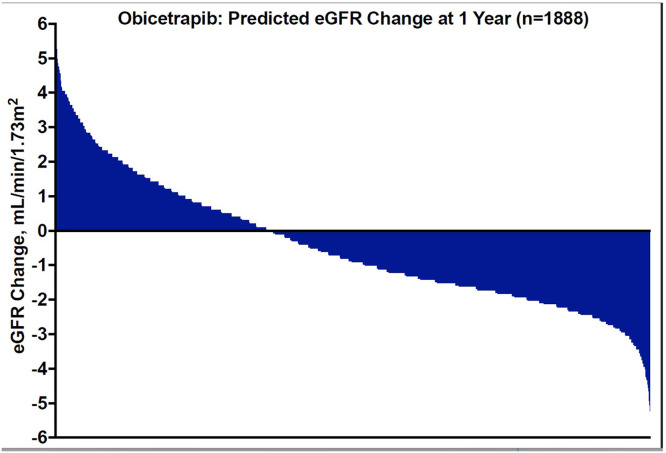
Obicetrapib: Predicted eGFR Change a 1 year (*n* = 1888).

Obicetrapib had a slower annualized decline in eGFR compared with placebo, and when eGFR assessments after the last dose of study drug were excluded (on-treatment analysis), there was an even larger difference. As evidence by waterfall plot analyses depiction, there was no evidence. Waterfall plots demonstrating individual-level eGFR change at one year for placebo and obicetrapib are shown in Fig. 2, Fig. 3 respectively. There was no evidence of heterogeneity in treatment effects by baseline eGFR <60 vs ≥ 60 mL/min/1.73 m^2^ for placebo (P_interaction_ = 0.44) or obicetrapib (P_interaction_ = 0.56), no significant difference was observed between groups in annualized changes in UACR.

On the basis of the baseline UACR stage (<20 mg/g, A1; 20–200 mg/g, A2; and >200 mg/g, A3/overt proteinuria), UACR mean (95 % CI) annualized placebo-adjusted percent changes were −3.48 % (−11.46 %, 5.22 %), −2.06 % (−17.99 %, 16.95 %), and −4.86 % (−43.94 %, 61.45 %), respectively.

**Fig. 4:**
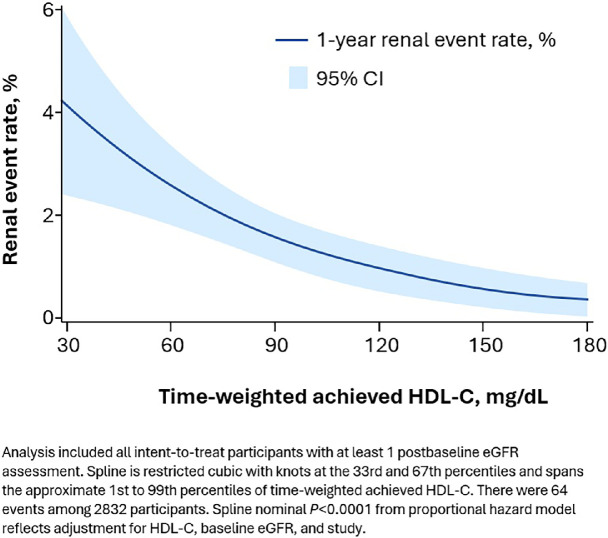
Spline of time-weighted achieved HDL-C vs the absolute risk of the renal event composite through 1year in BROADWAY and BROOKLYN.

Over a median follow-up of 1 year, participants receiving obicetrapib compared with placebo had nominally fewer composite renal events (36/1888 [1.9 %] vs (28/944 [3.0 %]), including fewer cardiovascular deaths (11/1888 [0.6 %] vs 8/944 [0.8 %]), fewer cases of eGFR <15 mL/min/1.73 m^2^ (0/1888 vs 2/944 [0.2 %]), and fewer cases of ≥40 % decline in eGFR (25/1888 [1.3 %] vs 18/944 [1.9 %]). There were no kidney transplants, dialysis initiations, or kidney-disease deaths in either treatment group. Taken together, the hazard ratio (HR) for the composite of renal events was 0.64 (95 % CI 0.39, 1.06), which was not statistically significant (nominal *P* = 0.08). The spline analysis of time-weighted achieved HDL-C vs renal event demonstrated a strong association between higher achieved HDL-C and lower renal event risk (spline nominal *P* < 0.0001), independent of baseline HDL-C and eGFR (Fig. 4).

## 4 Discussion

In this exploratory, post hoc pooled analysis of results from BROADWAY and BROOKLYN obicetrapib consistently demonstrated signals of attenuating renal function decline, including slower eGFR decline, fewer renal events, and a favorable trend in time-to-renal event analyses during a median 1 year of follow-up. The annualized decline in eGFR of 0.67 mL/min/1.73 m^2^ (nominal *P* value 0.02) should be recognized within the context of expected age-related decline as well as measurement variability. During normal healthy aging, kidneys have a progressive loss of function, with a median estimated decline in eGFR per year of ∼ 1 mL/min/1.73 m^2^ [14]. Additionally, obicetrapib’s renal composite HR of 0.64 (95 % CI 0.39–1.06; *P* = 0.08**)** is consistent with the modest renal effect sizes historically observed in non-renal populations receiving ACE-inhibitor–based therapy, where post-hoc analyses from HOPE, UKPDS, and ALLHAT showed small differences in GFR decline or renal event rates compared with other antihypertensive agents or placebo [[15], [16], [17], [18], [19]].

The strong association between higher achieved HDL-C and reduced renal risk, independent of baseline HDL-C and baseline eGFR, reinforces the biological plausibility of CETP inhibition as a reno-protective mechanism, potentially mediated through increased HDL, which would be expected to improve endothelial function, reduce inflammation, and enhance reverse cholesterol transport within the renal microvasculature [5,6]. Given that apoA1 is diminished in CKD and is critical for HDL's cholesterol efflux capacity, obicetrapib's substantial apoA1 elevation (+52.8 %) may be particularly relevant for restoring protective HDL function in this population. Additionally, obicetrapib increases pre-β₁ HDL [20], a particle characteristically abnormal in CKD owing to impaired HDL maturation and defective ATP-binding cassette transporter A1 (ABCA1)-dependent efflux [17]. These observations are consistent with drug-target Mendelian randomization evidence demonstrating that genetically lower CETP concentration is associated with reduced CKD risk [7]. However, our analysis from a 1-year intervention with an HDL-C raising agent cannot distinguish a causal effect and whether the signal detected reflects HDL biology, general drug response, or broader metabolic effects. There was no significant difference observed between groups in UACR changes. Although the effects were directionally favorable for obicetrapib, they were highly variable, resulting in an overall neutral effect. The absence of a significant effect on albuminuria, which is a key marker of renal injury, warrants further investigation. Notably, these findings emerged in a population with relatively preserved kidney function at baseline (mean eGFR 84–92 mL/min/1.73 m², median UACR 8–11 mg/g), suggesting that obicetrapib's renal effects may be detectable early, before significant nephropathy develops.

Because CETP inhibitors substantially raise HDL-C and elevated HDL-C is associated with reduced risk of ASCVD, the initial focus during the development of CETP inhibitors was on their HDL-C-raising effects [21]. That focus has now largely shifted to the ability of potent CETP inhibition to lower atherogenic apoB-containing lipoproteins thereby reducing ASCVD risk, as supported by evidence from animal models, observational cohorts, Mendelian randomization studies, and randomized controlled trials [22]. A pooled analysis of major adverse cardiovascular events (MACE) in BROADWAY and BROOKLYN indicated a lower, but not statistically significant, incidence of MACE (HR 0.77; 95 % CI 0.54, 1.11; *P* = 0.16), and a statistically significant reduction in coronary events (HR 0.68; 95 % CI 0.46, 1.00; *P* = 0.048) in participants treated with obicetrapib [13]. Furthermore, when the period beyond 6 months of randomization was examined alone, obicetrapib significantly reduced both cardiovascular (HR 0.60; 95 % CI 0.37, 0.99; *P* = 0.04) and coronary events (HR 0.45; 95 % CI 0.26, 0.77; *P* = 0.003). Importantly, similar to the association between HDL-C and renal events in the present analyses, higher achieved HDL-C was associated with lower risk of MACE.

While statins have a proven benefit for reducing cardiovascular events in CKD and may also reduce loss of renal function in earlier stages [18], as a class they only increase HDL-C by 5–10 %, far less than the 142 % elevation achieved with obicetrapib [8], and may not substantially affect apoA1 levels or HDL functionality, which are prominent features in the dyslipidemia of CKD. An examination of the relation between HDL-C and renal function in patients treated with atorvastatin demonstrated that higher HDL-C level was not robustly associated with improved eGFR, suggesting that increasing HDL-C with statins may not be a mechanism for improving renal function [23]. Treatment options to address suboptimal HDL in patients with CKD are needed [4].

Limitations of this post hoc analysis include the fact that the population had relatively preserved kidney function and low UACR at baseline, which limits the generalizability of these findings to patients at higher renal risk. Second, the median follow-up duration in both trials of 1 year was relatively short; renal progression is typically evaluated over longer timeframes. Third, the lack of adjustment for multiple endpoints increases the risk of a type I error. Fourth, because this was an exploratory, post hoc analysis of previously conducted trials for which renal outcomes was not a primary outcome of interest, these analyses were underpowered for assessing hard renal outcomes.

Our findings indicate that obicetrapib was associated with a modest attenuation in the eGFR decline over 1 year in a high cardiovascular risk population with largely preserved renal function consistent with the modest renal effect sizes historically observed in non-renal populations receiving ACE-inhibitor–based therapy, where post-hoc analyses from HOPE, UKPDS, and ALLHAT showed small differences in GFR decline or renal event rates compared with other antihypertensive agents or placebo [[15], [16], [17], [18], [19]]. Longer-term and appropriately powered studies are required to determine whether clinically meaningful renal benefit exists. Additionally, to our knowledge, similar renal signals with earlier CETP inhibitors have not been reported; however, torcetrapib's renal effects were confounded by off-target aldosterone-mediated blood pressure elevations [24], and dedicated renal analyses were not a focus of other CETP inhibitor programs. In a large population-based study, CETP genotype variation was not associated with differences in creatinine, eGFR, electrolytes, or blood pressure, consistent with renal neutrality of lifelong genetic CETP modulation [25]. There is also growing evidence to support the benefits of obicetrapib for raising HDL-C and improving HDL particle functionality in other conditions beyond ASCVD and CKD, including reducing new-onset type 2 diabetes mellitus [8] and preventing neurodegeneration [26].

**Figure:**
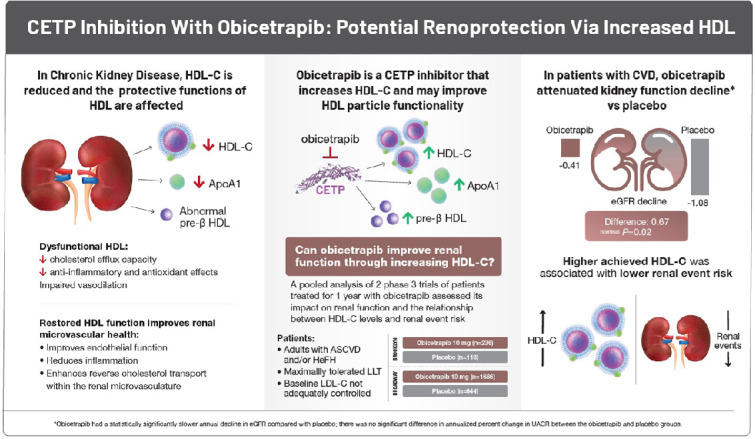
Central Illustration.

## Data statement

Data are available through the corresponding author upon reasonable request.

## CRediT authorship contribution statement

**Liffert Vogt:** Writing – review & editing, Conceptualization. **Michael H. Davidson:** Writing – review & editing. **Michael Szarek:** Methodology, Writing – review & editing. **Douglas Kling:** Writing – review & editing. **Mary R. Dicklin:** Writing – review & editing, Writing – original draft. **Danielle Curcio:** Writing – review & editing. **Marc Ditmarsch:** Writing – review & editing, Methodology. **Andrew Hsieh:** Conceptualization, Methodology, Writing – review & editing, Supervision. **Douglas L. Wicks:** Writing – review & editing. **Nancy Ortiz:** Writing – review & editing. **John J.P. Kastelein:** Conceptualization, Methodology, Writing – review & editing, Supervision.

## Declaration of competing interest

Liffert Vogt serves as a consultant for and/or has received research support, paid to his institution, from AstraZeneca, Bayer, Boehringer Ingelheim, CSL Vifor, NewAmsterdam, Novo Nordisk. MHD, DJ, DC, MD, AH, DW, NO and JJP are employees of NewAmsterdam Pharma and they also report that they receive stock or stock options. MRD: Employee, Midwest Biomedical Research, MS serves as a consultant for and/or has received research support from Lexicon, Amarin, NewAmsterdam, Silence, Sanofi, Regeneron, and Tourmaline. MS also receives salary support from CPC, a non-profit academic research organization affiliated with the University of Colorado, that receives or has received research grant/consulting funding between July 2021 and July 2024 from the following organizations: Abbott Laboratories, Agios Pharmaceuticals, Inc., Alexion Pharma Godo Kaisha, Amgen Inc., Anthos Therapeutics, Inc., ARCA biopharma, Inc., Arrowhead Pharmaceuticals, AstraZeneca Pharma India, AstraZeneca UK Ltd, Bayer, Bayer Aktiengesellschaft, Bayer Pharma AG, Beth Israel Deaconess Medical Center, Better Therapeutics, Boston Clinical Research Institute, LLC, Bristol-Myers Squibb, Cleerly, Inc., Colorado Dept of Public Health and Environment, Congress Inc, Cook Regentec LLC, CSL Behring LLC, Eidos Therapeutics, Inc., EPG Communication Holdings Ltd., Esperion Therapeutics, Inc, Faraday Pharmaceuticals, Inc., HeartFlow Inc, Insmed, Ionis Pharmaceuticals, IQVIA Inc., Janssen Pharmaceuticals, Inc, Janssen Research & Development, LLC, Janssen Scientific Affairs LLC, Lexicon Pharmaceuticals, Inc., Medpace, Inc., Medscape, Merck Sharp & Dohme Corp., Nectero Medical, Inc, Novartis Pharmaceuticals Corporation, Novo Nordisk Inc., Pfizer, PPD Development, L.P., Prothena Biosciences Limited, Regeneron, Regents of the University of Colorado (aka UCD), Sanifit Therapeutics S.A., Sanofi, Silence Therapeutics PLC, Stanford University, Stealth BioTherapeutics Inc., The Brigham and Women's Hospital, Thrombosis Research Institute, Tourmaline Bio, Inc, University of Colorado, University of Colorado Denver, University of Pittsburgh, VarmX, Verve Therapeutics, WraSer, LLC.

## Appendix Supplementary materials

Image, application 1
