# Supplementary material for: Cholesteryl ester transfer protein inhibition with obicetrapib is associated with attenuated decline in kidney function in patients at high cardiovascular risk: Post hoc pooled results from the BROADWAY and BROOKLYN trials
Source: Am J Prev Cardiol. 2026 Mar 24;27:101532. doi: 10.1016/j.ajpc.2026.101532 (PMC13261266; doi:10.1016/j.ajpc.2026.101532)
Supplement: Supplementary file 1 [file mmc1.docx]

**Table S1. Clinical characteristics and medication use at baseline**

| **Variable** | | **Obicetrapib**  **(N=1888)** | **Placebo**  **(N=944)** |
| --- | --- | --- | --- |
| Age, years | | 64 (11) | 64 (10) |
| Females, n (%) | | 680 (36.0) | 337 (35.7) |
| White race, n (%) | | 1437 (76.1) | 745 (78.9) |
| Body mass index, kg/m^2^ | | 29.4 (5.3) | 29.7 (5.7) |
| History of ASCVD, n (%) | | 1559 (82.6) | 778 (82.4) |
| Heterozygous FH, n (%) | | 506 (26.8) | 254 (26.9) |
| Diabetes, n (%) | | 658 (34.9) | 355 (37.6) |
| Hypertension, n (%) | | 1448 (76.7) | 738 (78.2) |
| eGFR, mL/min/1.73m^2^ | | 85.1 (18.2) | 85.7 (17.7) |
| HbA1c, % | | 6.2 (1.0) | 6.2 (1.0) |
| LDL-C, mg/dL | | 101 (39) | 101 (41) |
| HDL-C, mg/dL | | 50 (15) | 50 (15) |
| ApoB, mg/dL | | 93 (27) | 94 (28) |
| Triglyceride, mg/dL | | 122 (89, 170) | 127 (91, 175) |
| hsCRP, mg/L | | 1.3 (0.6, 3.4) | 1.4 (0.6, 3.2) |
| Medication use | |  |  |
|  | Statins, n (%) | 1712 (90.7) | 863 (91.4) |
|  | High intensity statins, n (%) | 1314 (69.6) | 648 (68.6) |
|  | Ezetimibe, n (%) | 573 (30.3) | 274 (29.0) |
|  | PCSK9 inhibitor, n (%) | 93 (4.9) | 59 (6.3) |

Table S1. Clinical characteristics and use of lipid lowering medications at baseline in patients treated with obicetrapib or placebo. Continuous variables presented as mean (standard deviation) or median (quartile 1, quartile 3) and categorical variables as number (percentage). Abbreviations: Apo, apolipoprotein; ASCVD, atherosclerotic cardiovascular disease; eGFR, estimated glomerular filtration rate; FH, familial hypercholesterolemia; HbA1c, hemoglobin A1c; HDL-C, high-density lipoprotein cholesterol; hsCRP, high-sensitivity C-reactive protein; LDL-C, low-density lipoprotein cholesterol; PCSK9, proprotein convertase subtilisin/kexin type 9.
